# Innovative Health and Social Integrated Care Model Effectiveness to Improve Quality Care for Chronic Patients: A Single Group Assignment Clinical Trial

**DOI:** 10.5334/ijic.6759

**Published:** 2023-10-11

**Authors:** Ester Gavaldà-Espelta, Maria del Mar Lleixà-Fortuño, Jordi Baucells-Lluis, Maria Ferré-Ferraté, Begoña Tomàs-Navarro, Claudia Curto-Romeu, Jorgina Lucas-Noll, Macarena Pozo Ariza, Elisabet Castro-Blanco, José Fernández Sáez, Carina Aguilar Martín, Alessandra Queiroga Gonçalves, Carmen Ferré-Grau

**Affiliations:** 1 Direcció d’Atenció Primària Terres de l’Ebre, Institut Catalàde la Salut, Tortosa; 2 Departament d’Infermeria, Programa de Doctorat Infermeria i Salut, Universitat Rovira i Virgili, Tarragona; 3 Campus Terres de l’Ebre, Universitat Rovira i Virgili, 43500 Tortosa, Tarragona, Spain; 4 Direcció de Serveis Territorials a les Terres de l’Ebre, Departament d’Igualtat i Feminismes, Generalitat de Catalunya, Tortosa; 5 Direcció de Sistemes d’Informació i Comunicació, Gerència Territorial Terres de l’Ebre, Institut Catalàde la Salut, Tortosa; 6 Equip d’Atenció Primària Amposta, Gerència Territorial Terres de l’Ebre, Institut Catalàde la Salut, Tortosa; 7 Unitat de Suport a la Recerca Terres de l’Ebre, Fundació Institut Universitari per a la Recerca a l’Atenció Primària de Salut Jordi Gol i Gurina (IDIAPJGol), Tortosa; 8 Unitat d’Avaluació, Direcció d’Atenció Primària Terres de l’Ebre, Institut Catalàde la Salut, Tortosa; 9 Unitat Docent de Medicina de Família i Comunitària, Tortosa-Terres de l’Ebre, Institut Catalàde la Salut, Tortosa

**Keywords:** integrated healthcare systems, chronic disease, quality of life, implementation science, information and communication technology (ICT)

## Abstract

**Background:**

Patients with chronic disease have become one of the major challenges for health and social protection systems in developed countries. Integrated care models (ICM) have demonstrably improved the quality of care of chronic patients. However, new models of integration need further evaluation of its effectiveness and outcomes.

**Methods:**

The ICM studied promoted coordination between the health and social sectors during a 6-month period, through an *ad hoc* developed application (app) that enabled a constant flow of communication between professionals from both sectors. Patients’ quality of life, treatment adherence, chronic patient experience and caregiver overload were assessed by questionnaires at baseline, at the end of the intervention and 6 months post-intervention.

**Results:**

The implementation of the new health and social ICM permitted new case detection and medical and social services offered to chronic patients. Furthermore, the quality of life and treatment adherence of patients and caregiver overload were significantly improved. These positive effects lasted at least 6 months after the intervention.

**Conclusions:**

Integrated care may facilitate access to care services, increase perceived patient quality of life and treatment adherence. Enhanced access to medical and social services from complex chronic patients may have important implications for caregivers and the care systems who are struggling to adapt to an expanding demand.

## Abstract (ca)

**Antecedents:**

Els pacients amb malalties cròniques representen un dels grans reptes per als sistemes de salut i protecció social als països desenvolupats. S’ha demostrat que l’atenció integrada millora la qualitat assistencial dels pacients crònics. Tanmateix, els resultats i l’efectivitat de nous models d’integració requereixen ser avaluats.

**Metodologia:**

El model d’atenció integrada d’estudi va promoure la coordinació entre els sectors sanitari i social durant un període de 6 mesos mitjançant una aplicació (app) desenvolupada ad hoc que permetia un flux constant de comunicació entre professionals d’ambdós sectors. La qualitat de vida dels pacients, l’adherència al tractament, l’experiència del pacient i la sobrecàrrega del cuidador es van avaluar mitjançant qüestionaris a l’inici, al final de la intervenció i 6 mesos després de la intervenció.

**Resultats:**

La implantació de l’atenció integrada social i sanitària va permetre la detecció de nous casos i un major accés dels pacients crònics als serveis mèdics i socials. A més, va millorar significativament la qualitat de vida i l’adherència al tractament dels pacients i la sobrecàrrega dels cuidadors. Aquests efectes positius van perdurar almenys 6 mesos després de la intervenció.

**Conclusions:**

L’atenció integrada pot facilitar l’accés als serveis assistencials, augmentar la qualitat de vida percebuda dels pacients i l’adherència al tractament. L’accés millorat als serveis mèdics i socials dels pacients crònics complexos pot tenir implicacions importants per als cuidadors i els sistemes assistencials que lluiten per adaptar-se a una demanda creixent.

## Introduction

Chronicity management is one of the main concerns of healthcare stakeholders and policymakers in developed countries. In the EU, the population over 80 years will grow from 5% in 2010 to 11% by 2050 [1]. Chronic disease is strongly associated with poor quality of life and functional status, higher rates of health service use, and greater costs and patient and caregiver burden. Around 30% of patients aged over 65 years of age have multi-morbidity, presenting more than five chronic conditions [2]. However, most chronic diseases display similar demands on healthcare and social systems regardless of etiology [3], meaning that the central question is how we can adapt healthcare systems to achieve the best welfare outcomes, rather than addressing multi-pathological segmented assistance [4]. To this end, evidence-based strategies should be implemented to build new, multidisciplinary and inter-sectoral paradigms that are adapted to contemporary needs [5].

Person-centered and integrated care models such as the Expanded Chronic Care Model (ECCM) and the Innovative Care for Chronic Conditions (ICCC) aimed to build clear associations between the healthcare system and the community. By strengthening community action, creating supportive environments and keeping patients informed it is promoted the self-management and personal skills that allow the re-orientation of health services to build public health policies and stimulate proactive community partners [6,7]. These innovative approaches for managing chronic conditions have had a variety of positive effects on health and social outcomes, considering that these strategies embrace the intrinsic role of social determinants of health [8]. Thus, there is evidence of a clear improvement in biological disease indicators, a reduction of deaths, quality of care and patient satisfaction, self-management abilities, improvement in function and quality of life and greater effectiveness in care-managing processes and costs [9,10,11,12,13]. Despite the robustness and growing evidence advocating for innovative integrated care models, the embrace of the healthcare and social service workforce, patients and families remains a challenge for healthcare stakeholders in Europe and globally, which mostly remain fragmented and designed to solve single, acute and short-term diseases [14].

The purpose of the present study was to boost the Catalan Health Strategy Plan [15] and to drive evidence into practice through the development, implementation and evaluation of an innovative integrated care model directed at chronic patients and their caregivers. The Health Strategy Plan is the instrument guiding all public health policies of the Government of Catalonia and for the 2016–2020 period developed two specific programs regarding chronicity assistance. The Chronicity and Prevention Care Program (CPCP; PPAC in Catalan acronyms) which was an initial individualized care program mainly committed at defining criteria of patients with chronic conditions and complex needs together with defining protocols for the screening and detection of these patients [16]. Secondly, the Health and Social Interaction and Care Interdepartmental Plan (HSICIP; PIAISS in Catalan acronyms) which defined for first time “a model based on a health and social integrated care, person-centered that guarantees the continuum of care and efficiency in the use of resources”. This program also ascertains the requirement of promotion, monitoring and evaluation of bottom-up designed territorial projects and the need of the ICT tools development to satisfy the program demands [17]. These programs were the first highly effortful intent to adapt to the abovementioned demographic transition towards an abovementioned increasing aged and multimorbid population, since healthcare and social systems were not created and developed to fit the actual chronic patients’ needs becoming costly and resourceful highly inefficient. Thus, in this context we pursued the integration of health and social services together with the active participation of patients and their caregivers to implement integrated care using strategies of professional training, patients’ educational outreach visits and a new ICT tool [18]. Concretely, we promoted a horizontal integration of existing health and social services through multidisciplinary teams composed by professionals of health and social sectors and by an informatic system that registers and integrates all the cases information and actions performed. Previously, this information was atomized and inaccessible by the different professionals causing duplicity of tasks by professionals and excessive patients’ visits and processes requests. The proposed integrated care model implies the knowledge by all professionals involved of service portfolio of all care provider partners, a new competence distribution to achieve real coordination between sectors, provide new governance roles and display a proactive attitude towards patients, caregivers and families to accomplish a successful integrated care in detriment to the conventional procedures and assistance protocols.

The main objective of this study was to evaluate the effectiveness of a new integrated care model in patients with chronic conditions in a real context with the existing local available resources and workforce. The primary outcome of the study was patients’ quality of life. The measurement of quality of life is increasingly important to both the health and social care services [19]. As a subjective multidimensional concept, it helps capture and evaluate broad aspects of life, including the social determinants of health, which is a key aspect to consider in the design and implementation of integrated care systems.

## Methods

Implementation and outcome assessment were carried out following a previously described clinical trial study protocol [18] and the Medical Research Council guidelines on the evaluation of complex interventions [20]. The Template for Intervention Description and Replication (TIDIeR) checklist was used to guide reporting of the present study [21]. Herein we present the results of effectiveness of the intervention.

### Trial design

**Figure 1:**
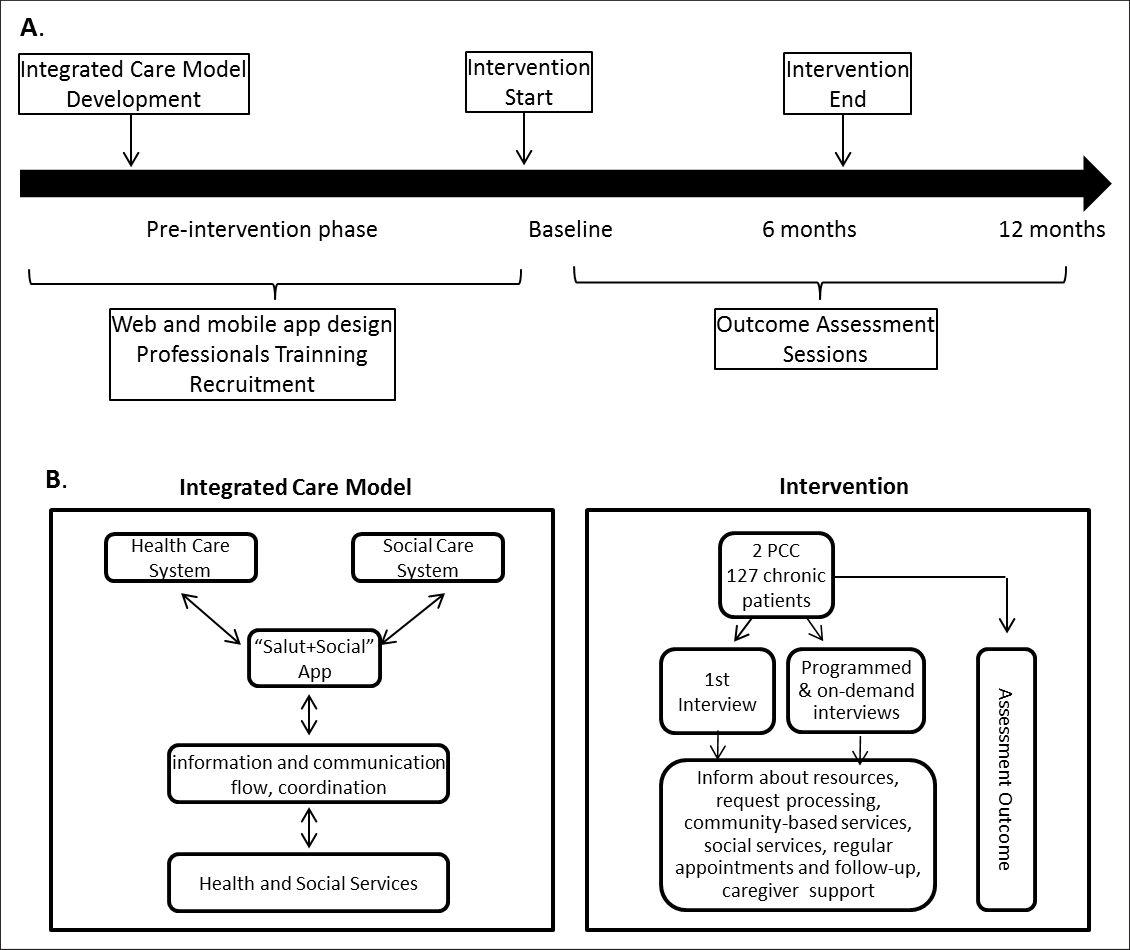
***Salut+Social* integrated care model implementation and intervention plan. A**. Study design flowchart. **B.** Integrated care model (left) and Intervention (right) schemes.

A quasi-experimental clinical trial based on a multicenter single group assignment intervention was developed. Pre and post repeated measures evaluated the effect of the implementation of a new integrated care model on chronic patients’ quality of life, treatment adherence, chronic patient experience and caregiver burden. For sample size calculation for the primary outcome (patients’ quality of life) we aimed to obtain a difference of at least 0.1 units (0.25 SD) in the EQ-5D-3L questionnaire. For that, we assumed an alpha error of 0.05 (95% confidence interval), a beta error of 0.10 (90% power) in a bilateral contrast and a 45% loss to follow-up. The formulas used were: n = (Zα·Zβ)^2^·S^2^/d^2^ and n_final_ = n_initial_·(1/1–R). Accordingly, we estimated a minimum sample size of 120 subjects. The study spanned 1 year, with the intervention being carried out over 6 months, from June to November 2019. At June 2019, the baseline sessions were performed, and follow-up measurement sessions were conducted at the end of the intervention and 6 months post-intervention (Figure 1A). The study protocol was reviewed and approved by the Clinical Research Ethics Committee of the *Fundació Institut Universitari per a la Recerca a l’Atenció Primària de Salut Jordi Gol i Gurina* (code P17/100) and registered on Clinicaltrials.gov (Identifier: NCT04164160; November 15, 2019).

### Setting and participants

A single group of chronic and social-dependent patients (See Box 1 for disease and social condition definition) were recruited from two primary care centers (PCCs) of the Catalan Health Institute (CHI) from the *Terres de l’Ebre* region of Catalonia.

**Box 1 Health and social condition definitions.**

**Complex chronic patient:** Patient requiring a special assistance plan due to a complex clinical condition management, generally for accumulation of concurrent chronic diseases, accompanied by an intensive resource use, especially hospital admissions that may be avoided.

**Advanced chronic disease:** Patient with at least one chronic disease in a critical, advanced or intensifying phase, with added assistance needs and limited life prognosis.

**Home care program:** Patients who due to their health status and community or organizational characteristics require regular home assistance to perform basic activities of daily living (ADL) for a particular period of time.

**Dependence degree 1:** Patient requiring assistance recurrently, or at least once a day.

**Dependence degree 2:** Patient requiring assistance twice or thrice a day, but without requiring a permanent caregiver.

**Dependence degree 3:** Patient requiring assistance throughout the whole day to perform basic ADL.

**Home assistance:** Patient with limited level of autonomy requiring either assistance with household needs (cleaning, cooking, shopping, laundry, etc.) or personal care (hygiene, dressing, etc.)

**Teleassistance:** Phone assistance 24/7. Patient with limited level of autonomy requiring phone assistance for emergencies, medical, familiar or other alerts, recurrent monitoring of activity, etc.

*Inclusion criteria* (all of which criteria had to be met):

- Adult patients with at least with one health and/or one social condition, as specified:
  - Health condition: Complex chronic patient, advanced chronic disease, patient in the home care program, dementia, neurodegenerative disease, stroke, other chronic disease.
  - Social condition: Dependence, home assistance, teleassistance [22,23].
- Knowledge of Spanish or Catalan.
- Acceptance of participation in the study (informed consent signed by the patient).

*Exclusion criteria* (any of which criteria could be met):

- Institutionalized patients.
- Users with difficulties filling out responding to questionnaires.

Patients’ caregivers participated as study subjects for overload evaluation.

*Inclusion criteria* (all of which criteria had to be met):

- Meet Primary Caregiver definition: “*person identified in an individual support plan as providing the majority of service and support for an individual in the individual’s home*
- Acceptance of participation in the study (informed consent signed by the caregiver).

### Intervention

The intervention consisted of promoting the coordination of health and social care services targeting patients with chronic or social dependence conditions by means of a new ICT tool that creates an interface for the implementation of a new integrated care model, named *Salut+Social*. The ICT tool consists of a web and mobile app (*Salut+Social* app), feed with data from the electronic clinical history of the CHI and social data from social services workstation from the studied district, that allows a constant communication flow between participating professionals from both sectors (nurses, general practitioners (GP) and community-based social workers).

Participating professionals were instructed and trained before starting the study to understand each other’s service portfolio, the multidisciplinary approach to chronic patient management and the use of the *Salut+Social* App. As a first step, the *Salut+Social* app facilitated the identification and selection of patients who met the inclusion criteria since their clinical and social data were automatically entered into the app, from health and social electronic databases, ECAP and Hestia, respectively. Next, patients and caregivers were contacted to arrange a first interview, in which they were informed about the new integrated care model and coordinated actions. The main actions promoted were: to provide information about available resources and grants for chronic patients, information and processing requests on community-based and social-dependence services, regular appointments and follow-up with medical and social services, and health advice for caregivers. In the first interview, patients and caregivers responded to the study questionnaires and participated in planning the new care. During the intervention, information was constantly updated in *Salut+Social* app and multidisciplinary visits could be requested by any professional whenever they were considered necessary (Figure 1B). Patients and caregivers received an appointment to attend their PCC 6 and 12 months after their incorporation into the program and were asked to respond to study questionnaires.

### Data collection and outcome assessment

Patients’ sociodemographic and caregiver characteristics were evaluated by *ad hoc* questionnaires. The main outcome of implementing the intervention is the health-related quality of life of the target population, patients with chronic and social dependence condition, assessed by the EQ-5D-3L questionnaire [24,25]. Secondary outcomes related to the integrated care intervention effectiveness, such as treatment adherence, chronic patient experience and caregiver overload, were assessed by Morisky-Green [26,27], IEXPAC [28] and Zarit [29,30] questionnaires, respectively. All the questionnaires were filled in baseline and follow-up sessions, except for IEXPAC that was filled in follow-ups. Social service requests and benefits were registered in the *Salut+Social* app throughout the intervention and subsequently evaluated. The number of activities and coordinated actions carried out and recorded in the *Salut+Social* app were collected and quantified at the end of the study.

### Statistical analysis

Univariate summaries of all investigated variables are presented as means with standard deviation (SD) or frequencies and percentages for quantitative and categorical variables, respectively. To evaluate the effect of the intervention on the scores obtained in the specific questionnaires (EQ-5D-3L, IEXPAC and Zarit test) and the distribution of patients with or without treatment adherence, or with social services assignment, or not, throughout the study, paired and unpaired comparisons of frequencies and means comparisons were carried out to assess the effects on categorical or quantitative variables before and after the intervention, respectively.

To evaluate the modeling effect of subject variables on the effectiveness of the intervention for assignment of hours of care and dependency, a mixed linear and a logistic generalized regression model was employed, respectively. To assess the effect of the level of severity of the various chronic diseases on the dependent variables studied, a new variable named ‘degree of morbidity’ was derived. Three degrees of morbidity were defined: ‘low morbidity’ which included stroke patients; ‘medium morbidity’ which included complex chronic patients; and ‘maximum morbidity’ that comprised patients with advanced chronicity, neurodegeneration, and dementia. Data were nested using a subject-specific random intercept so that data are clustered within subjects from baseline to 6 and 12 months post-intervention. The subject variables of age, sex and degree of morbidity were treated as fixed effects. The effect of the subject’s variables on intervention fidelity is estimated by the beta regression coefficient (with SD) for hours of care received and by the odds ratio (OR) with 95% CI for the binary outcome assignment of dependency vs. non-dependency.

To examine the effect of the intervention on health-related quality of life measured by the EQ-5D-3L scores, a generalized linear regression mixed model was developed. The data were nested as previously described. The subject variables of age, sex and degree of morbidity were treated as fixed effects. The social variables of dependency and hours of care received were included as fixed covariates in the model. Statistical tests used are specified in the figure legends.

For all statistical tests significance was concluded for values of p < 0.05, with a 95% CI. Data were analyzed using R 4.0.2. and Prism 8.

## Results

### Patient and caregiver demographic characteristics

Of the 1132 eligible patients screened from the 2 PCCs of the study, 287 (25%) were found to meet the inclusion criteria and were included in the study. A loss to follow-up of 27% was recorded by 6 months and of 44% by 12 months. Most loss was due to institutionalization and *exitus*. Consequently, 127 (56%) of the 227 patients participated fully in the study. The sample was unbalanced with respect to gender (65% women and 35% men), although this reflects the ratio in the global population of Catalonia, in which the proportion of women with chronic disease is higher that of men [31]. The mean age was 79.59 (15.91) years. Overall, the majority of patients were retired (83%) and only about 35% had a partner at the time of the intervention. The sample represented a broad population of patients with chronic disease conditions: mainly chronic complex patients (49%), home care program patients (41%), patients with dementia (17%) and stroke (16%) (Table 1). The majority of caregivers were women. Most were patients’ relatives, either the offspring (54%) or the partner (23%). Only 10% were remunerated caregivers (Table 1).

**Table 1:** Demographic characteristics of patients and caregivers.


PATIENTS	N	%

All patients	127	100

**SEX**	**N**	**%**

Female	82	65

Male	45	35

**AGE**	**MEAN**	**SD**

Age	79.59	15.91

**CIVIL STATUS**	**N**	**%**

Widow/er	58	46

Married	45	35

Single	22	17

Divorced	2	2

**EMPLOYMENT**	**N**	**%**

Retired	105	83

Disabled	17	13

**EDUCATION LEVEL**	**N**	**%**

Illiterate	10	8

Primary education	111	87

Secondary education	5	4

Higher education	1	1

**HEALTH CONDITION**	**N**	**%**

Complex chronicity	62	49

Home care program patient	52	41

Dementia	21	17

Stroke	20	16

Neurodegeneration	12	9

Advanced chronicity	5	4

**CAREGIVERS**	**N**	**%**

All caregivers	122	100

**KINSHIP**	**N**	**%**

Offspring	58	48

Partner	28	23

Parents	7	6

Grandparents	1	1

Brother/Sister	4	3

Niece/Nephew	7	6

Grandchildren	1	1

Son/Daughter-in-law	5	4

**REMUNERATED**	**N**	**%**

Yes	10	8


### Participating professionals’ characteristics and compliance with the intervention

Participating professionals were mostly primary care (PC) nurses and general practitioners, all professionals combined their involvement in the intervention with their usual daily work activity except for one PC nurse, who carried out the main tasks of promotion, training and coordination of the other participating professionals with full-time dedication to the intervention (Table 2). Implementation of the intervention achieved a good compliance and fidelity whereby >1000 activities were recorded between professionals from both sectors. In fact, the flow of information between the health and social sectors had been totally bidirectional and with equal participation of the sectors. Of the different coordinated activities carried out, the number of new cases detected and registered (>500) and the number of patient follow-up activities (>250) were of note. Patients who did not meet the study’s inclusion criteria were excluded from the intervention but were included in the *Salut+Social* program so that as many chronic patients as possible could benefit. It should also be noted that the most active professionals throughout the intervention were PC nurses and community-based social workers (Table 2).

**Table 2:** Professionals’ characteristics and degree of compliance with intervention.


PROFESSIONALS	N	%

All professionals	58	100

**PROFESSIONAL PROFILE**	**N**	**%**

Primary care nurse	19	33

General practitioner	13	22

Health referee	10	17

Social referee	4	7

Social worker (community)	9	16

Social worker (hospital)	2	3

Social educator	1	2

**DEDICATION TO INTERVENTION**	**N**	**%**

Full-time	1	2

Part-time	57	98

**COORDINATED ACTIVITIES (INSIDE THE INTERVENTION)**	**N**	**%**

Patients followed-up	257	91

Joint home visit	24	8.5

Joint interviews	1	0.5

**COORDINATED ACTIVITIES (INSIDE AND OUTSIDE THE INTERVENTION)**	**N**	**%**

New case registration	544	88

Joint meetings	74	12

***SALUT+SOCIAL* APP USAGE**	**N**	**%**

All activities	1005	100

**ACTIVITIES BY DIRECTIONALITY**	**N**	**%**

From Health to Social	513	51

From Social to Health	492	49

**ACTIVITIES BY PROFESSIONAL PROFILE**	**N**	**%**

Primary care nurse	238	23

General practitioner	89	9

Health referee	167	17

Social referee	268	27

Social worker (community)	190	19

Social worker (hospital)	19	2

Social educator	34	3


### Patient health and social outcomes and caregiver overload

**Figure 2:**
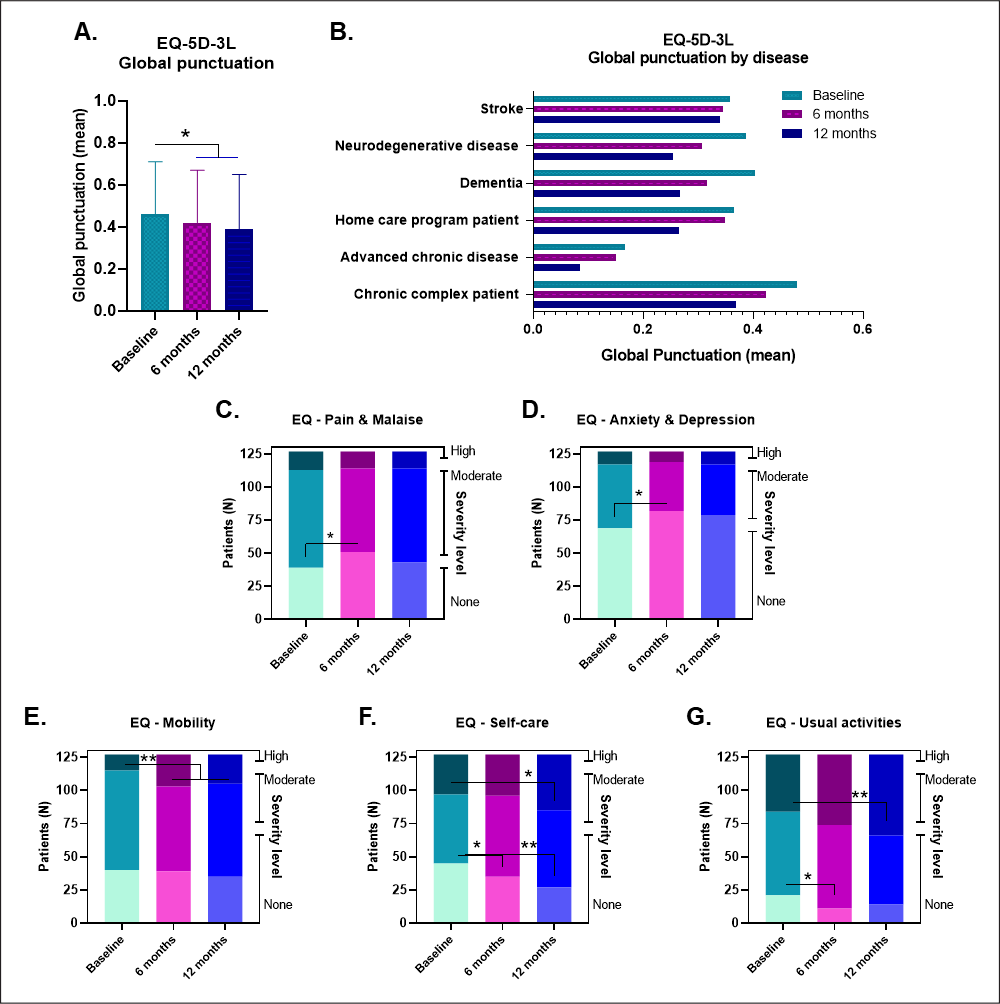
**Effect of the *Salut+Social* intervention on patients’ perceived health-related quality of life**. **A.** EQ-5D-3L global punctuation (mean ± SD) at baseline, 6 months and 12 months after the initiation of the intervention. **B.** EQ-5D-3L global punctuation (mean) by health condition at baseline, 6 months and 12 months after the start of the intervention. **C-G.** Proportion of patients (n) at each severity level in the dimensions of pain and malaise (**C**), anxiety and depression (**D**), mobility (**E**), self-care (**F**) and usual activities (**G**) of the EQ-5D-3L at different times. Statistical analysis was by Student’s t test in (A) and McNemar’s test in (C-G). * p < 0.05, ** p < 0.01.

The *Salut+Social* integrated care model intervention aimed to improve the quality of life of chronic patients. The EQ-5D-3L is a descriptive survey comprising five dimensions: mobility, self-care, usual activities, pain and discomfort, and anxiety and depression. Each dimension spans three levels of severity: null, moderate or high. Results are expressed either by calculating the Global punctuation, being 1 the optimal health status value (Figure 2A and 2B), or by presenting the descriptive data by dimensions (Figure 2C, D, E, F, G).

Before the intervention, the mean EQ-5D-3L score was 0.46 (0.25), indicating a low basal health status of chronic patients that actually slightly declined over time (Figure 2A). Within the sample studied, patients with advanced chronic disease showed the worst health-related quality of life at baseline and throughout the study (Figure 2B). The descriptive analysis by dimension revealed that the proportion of patients with a null severity level specifically in the dimensions of pain or discomfort and anxiety or depression significantly increased after the intervention. Consequently, the proportion of patients with moderate pain and/or depression at the beginning of the study decreased at the end. Patients with high intensity in these dimensions did not change during the intervention (Figure 2C and 2D). It is of particular note that these results showed how chronic diseases are highly detrimental to the functionality and physical abilities of patients regarding mobility, self-care and usual activities (Figure 2C, D, E, F, G), but, surprisingly, the integrated care program was able to improve the emotional and psychological dimensions of these patients.

**Figure 3:**
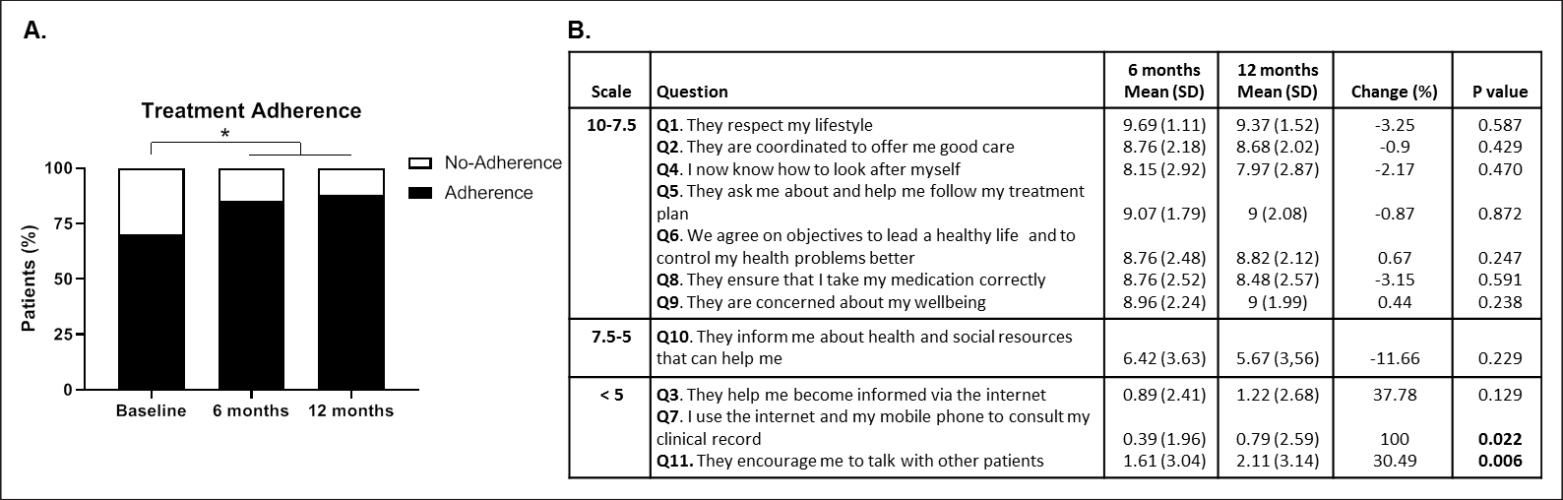
**Effect of the *Salut+Social* intervention on patients’ treatment adherence and chronic patient experience**. **A.** Percentage of patients (%) showing appropriate treatment adherence according to the Morsiky-Green dichotomous questionnaire. **B.** Mean score (SD) obtained for each question of the IEXPAC questionnaire 6 and 12 months after the beginning of the intervention. Statistical analysis was by McNemar test in (A) and Wilcoxon test in (B).

To further evaluate the effectiveness of the intervention in the quality of care of chronic patients, medication adherence was measured. It is a good proxy for proper care as these patients usually need long-term therapies that require persistence, vigilance and motivation. The proportion of patients with adequate treatment adherence increased after the intervention and this increment was maintained until the end of the study, 6 months after the intervention had finished, achieving a reduction in the percentage of patients without good treatment adherence to only 12% of all chronic patients compared with 30% at baseline (Figure 3A). This suggests that the integrated model provided the support and habit formation required for chronic patients’ appropriate medication over the long term.

Furthermore, the quality of care of chronic patients received by health and social services under the new integrated care model was evaluated through the IEXPAC questionnaire, which allows the degree of coordination and the quality of the attention provided by the professionals to be audited. The IEXPAC consists of 11 questions assessed on a scale from 0 to 10. Notably, most questions yielded high scores (>7.5) (Figure 3B), suggesting an overall outstanding quality of care after the intervention. Curiously, the items regarding new models of relationship, including new sources of information, access to information through the Internet and support from other patients and the community achieved the lowest scores, <5 but were those that significantly improved by the end of the study (questions 7 and 11) (Figure 3B).

**Figure 4:**
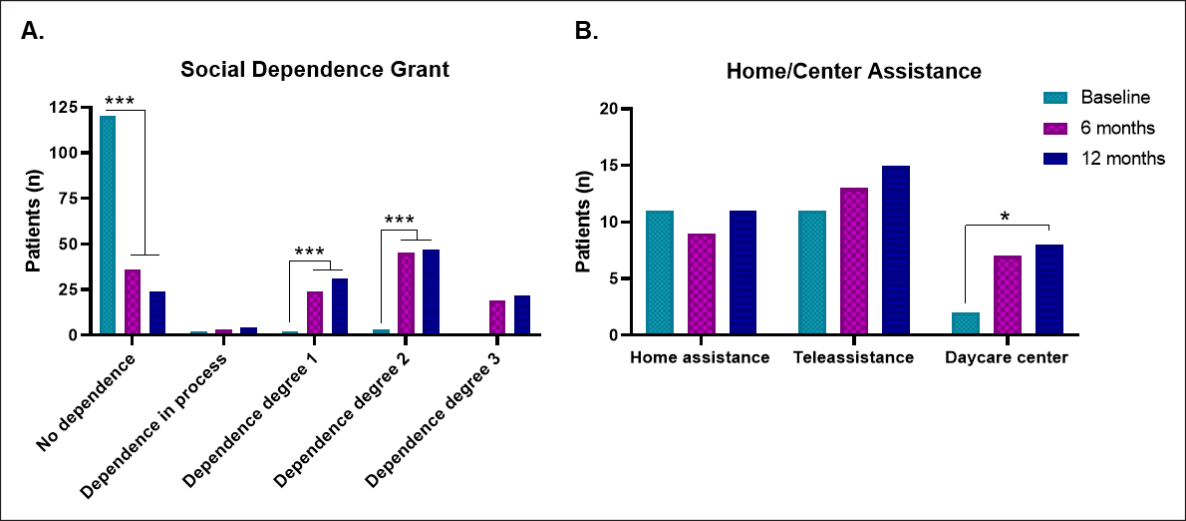
**Effect of the *Salut+Social* intervention on patients’ engagement with social services**. **A.** Proportion of patients (n) with different grades of social dependency assignment. **B.** Proportion of patients (n) with home assistance or daycare center assignment. Statistical analysis involved the McNemar test in (A) and (B). *p < 0.05, ***p < 0.001.

Managing chronic conditions often requires attendance from social services, since health services alone are not sufficient to cover and guarantee the wellbeing and care needs of chronic patients. For this reason, degree of dependency and the different home assistance modalities and social services were registered on the *Salut+Social* app throughout the intervention and subsequently evaluated. As a result, the intervention promoted and favored the request and granting of social dependency recognition of grades 1 and 2. Thus, the proportion of patients without recognized social dependency significantly decreased (75%) after the intervention (Figure 4A) and patients granted social dependency grades 1 and 2 increased (23% and 35%, respectively) compared to baseline (p < 0.001) (Figure 4A). Regarding the social services of home assistance and teleassistance, no changes related to the intervention were observed (Figure 4B). In contrast, 6% of the sample obtained an allocation at a daycare center (Figure 4B).

**Figure 5:**
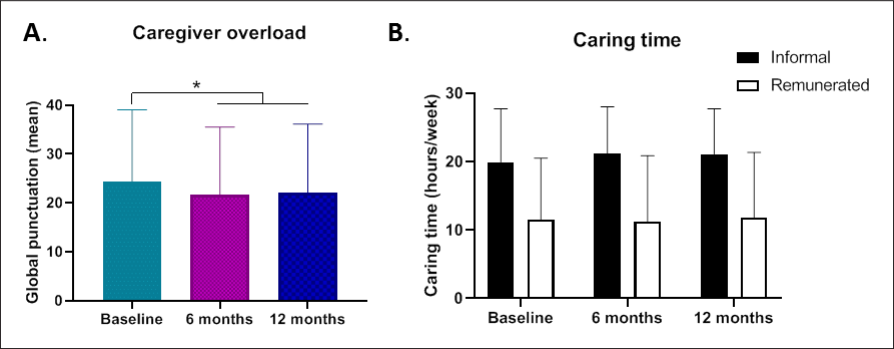
**Effect of the *Salut+Social* intervention on caregiver overload**. **A.** Mean (SD) caregiver burden score according to Zarit test at baseline and 6 and 12 months post-intervention. **B.** Mean (SD) hours/week of informal and remunerated caregivers’ time spent in caring. Statistical analysis was by McNemar test in (A); *p < 0.05.

*Salut+Social* model is mainly a patient-centered model but is also a caregiver-centered approach since for patients with chronic conditions, the assistance of their caregivers is a fundamental determinant of their health status and quality of life. Thus, new models of chronicity care need to take into consideration caregivers’ overload and provide resources and mechanisms to guarantee the wellbeing and optimal conditions for long-term care support. Caregiver burden was assessed before and after the intervention using the Zarit test. Remarkably, the mean score obtained at baseline was 24.36 (14.64), which reflects that some caregivers clearly suffered overload (>17) before the intervention (Figure 5A). After the intervention, a 2-point decrease in caregiver burden was observed, and this was maintained until the end of the study, 6 months post-intervention (Figure 5A), supporting the notion that the integrated care program provided resources to patient care that had a beneficial effect on caregivers. Interestingly, the improvement in caregivers’ burden was not due to a reduction in the time dedicated to care, but probably to the quality of this dedication, since the time spent in caring did not change throughout the study, with values of 19.97 (7.77) hours/week for informal caregivers and 11.55 (8.97) hours/week for remunerated caregivers over time (Figure 5B).

Finally, to assess the moderating effect of sociodemographic characteristics and the degree of exposure to the intervention on the health-related quality of life of chronic patients, a multivariate analysis was performed. First, we studied which subject variables were related to a greater or lesser assignment of dependency and hours of care. Table 3 shows that age is positively correlated with the hours of care (Beta > 0) of chronic patients received by their caregivers, while it is negatively related to the dependency assignment (OR < 1). As this negative relationship between age and dependency was unexpected, it was further analyzed using a contingency table with the mean age of patients with or without dependency throughout the study. As described above, before the intervention most of the patients had not recognized any degree of dependency (Figure 3). Their mean age was 80.2 (15.38) years, while at this time the mean age of patients with some degree of dependency was 64.8 (23.08) years. After the intervention, the mean age of dependent patients was 76.95 (17.20) and 78 (16.32) years at 6 and 12 months post-intervention, respectively; by contrast, patients without dependency remained at 85.54 (10.49) and 85.18 (13.14) years, respectively. These results show how the average age of dependent patients increased by 15 years by the end of the intervention. Sex is not determinant for dependency assignment or hours of care received. However, the degree of morbidity was strongly positively associated with these parameters. Specifically, presenting a maximum degree of morbidity is associated with a 4.2-times greater probability of obtaining dependency and with 4 hours more weekly care. Conversely, patients with a medium degree of morbidity were not statistically more likely to obtain dependency, but did obtain more hours of care (beta = 3.03, p = 0.035) (Table 3).

**Table 3:** Moderating effect of patients’ sociodemographic characteristics on the grade of intervention’s exposure.


	DEPENDENCY OR (95% CI)	P VALUE	HOURS OF CARE BETA (SD)	P VALUE

Age	0.94 (0.91–0.98)	**0.001**	0.12 (0.04)	**0.008**

Sex (Male)	0.89 (0.30–2.58)	0.826	1.18 (1.48)	0.426

Medium-grade morbidity	0.62 (0.19–2.03)	0.426	3.03 (1.43)	**0.035**

Maximum-grade morbidity	4.20 (1.01–17.41)	**0.048**	4.41 (1.75)	**0.012**


According to the estimates of the adjusted multivariate model, patients suffering from advanced chronic disease, neurodegeneration or dementia (maximum degree of morbidity) experience a reduction in their health-related quality of life of 0.13, 0.11 and 0.08 relative to the mean score of the EQ-5D-3L, respectively, compared with stroke patients (low degree of morbidity) (p < 0.1). However, the variables of the intervention dependency and hours of care were negatively associated with the EQ-5D-3L score, indicating that patients with greater deterioration in their health-related quality of life received greater assignment of services and care by their caregivers throughout the intervention (p < 0.01 and p < 0.001) (Table 4). Overall, the results of the multivariate analysis suggests that the health-related quality of life of chronic patients was strongly influenced by the level of severity of their disease, as well as that the integrated care intervention took into consideration the needs of the chronic patients, showing a high adherence so that the available resources were assigned to patients with greater functional deterioration.

**Table 4:** Effect of patients’ sociodemographic characteristics and exposure to the intervention on health-related quality of life.


	EQ-5D-3L BETA (SD)	P VALUE

Intercept	0.71 (0.09)	**<0.001**

Age	–0.001 (0.001)	0.322

Sex (men)	0.055 (0.039)	0.163

Stroke	–0.071 (0.048)	0.141

Complex chronic patient	–0.042 (0.034)	0.223

Advanced chronic disease	–0.136 (0.079)	0.086

Neurodegeneration	–0.117 (0.066)	0.076

Dementia	–0.084 (0.050)	0.094

Dependency	–0.068 (0.019)	**0.009**

Hours of care	–0.006 (0.002)	**<0.001**


## Discussion

The main contributions of this study to the scientific evidence about the management and care of complex chronic patients are, on the one hand, the *Salut+Social* integrated care intervention, which was based on the development and use of a web and mobile app that facilitates integration of the health and social sectors, and, on the other, the data obtained that demonstrate how this type of integrated care for chronicity is effective in: 1) newly detected cases who are suitable for incorporation into the integrated care program; 2) the improvement in the quality of care of these patients, reflected by the improvement in 2a) the perceived health-related quality of life regarding emotional and psychological wellbeing, and 2b) adherence to treatment. Finally, there was also an effective reduction in the long-term caregiver burden. These data are highly relevant since they have a direct impact on a highly demanding and growing sector of population that is highly vulnerable [31]. Similarly, health and social service delivery systems are also highly vulnerable due to a lack of resources and an elevated attendance pressure.

This study is unique in the field of integrating PC and social services in Catalonia and Spain since, despite the growing initiatives of the different regional health service systems that are starting to implement integrated care models, especially Andalusia, the Basque Country and the Valencian community, no exhaustive effectiveness evaluations have been carried out at scientific level [32]. In contrast, a recent systematic review compared the studies of integrated care interventions in the United Kingdom and Europe (9) and highlighted that the different integration models are mainly evaluated in relation to: the use of health care resources, the quality of care received by patients, and the work experience of professionals exclusively. All these parameters were evaluated simultaneously in this study. The most robust results obtained in the meta-analysis were in the same direction as those obtained in this study, indicating that: first, basically, integrated care leads to greater patient satisfaction; second, integration increases the perceived quality of care received; and third, integration increases chronic patients’ access to health and social services. Likewise, it emphasizes the need for more studies to evaluate the implementation of the various integrated care programs that have already been implemented, since despite being a great consensus on what is needed for care integration, there is still a knowledge gap concerning the best integration model according to the context and the resources [33,34].

The *Salut+Social* intervention achieved a highly integration and coordination of PC, hospital and social services with absolutely no previous connection through the training of professionals in the multidisciplinary integration model and the use of the innovative app developed *ex profeso*. In fact, these were the key aspects for the sustainability of the model in routine practice. Professionals’ engagement and the fitted app embracing the needs of both sectors. To assure a sufficient professionals’ knowledge and commitment with the model, the health and social referent figure was essential, making it indispensable to incorporate these new professional roles to guarantee the sustainability of the model. Nonetheless, according to the qualitative study performed after the intervention [35], professionals requested standardized protocols and the integration of the app in their routine electronic platforms. The qualitative study helped us to identify the elements of the model to improve, however, it potentially could be implemented in other communities for health and social services integration.

Regarding the health outcomes, an improvement in the health-related quality of life was obtained specifically in the dimensions related to emotional wellbeing, such as the perception of pain or discomfort and anxiety or depression, in accordance with previously published results that also showed how integrated care in chronic patients is able of to improve specific aspects of health-related quality of life to the detriment of absolute and total improvement [36]. Regarding the quality of care received by patients with the new integrated care model, it should be noted that after the intervention most of the elements were very positively evaluated, reflecting a high quality of patient care systems. However, it also highlights which elements of the system are the weakest. These were the elements related to patients’ capacity to self-inform autonomously and relate socially or with other patients. In this sense, integrated care is clearly directed towards improving these elements, showing a lasting effect on the patients, leading us to reinforce these aspects of the intervention in terms of implementation expansion phases of the model. The *Salut+Social* intervention not only had positive effects on patients’ subjective perception of their health-related quality of life or care received, but also improved the degree of adherence to their treatment and therapies, which probably contributes not only to their wellbeing and better long-term prognosis, but also to preventing the deterioration or progression of the disease that avoids acute admissions and additional services over the long-term. Thus, more studies are needed to evaluate the effectiveness of integrated care in determining the frequency of avoidable PC visits, hospital admissions and emergency care [37]. Another particular, key factor of the *Salut+Social* intervention was the evaluation of the burden on caregivers of chronic patients. This aspect has been poorly addressed to date by the scientific literature. Although the intervention did not affect the caring time required by patients from their caregivers, the caregivers’ burden decreased significantly due to the support received from professionals.

From a gender perspective, it is of note that although no significant differences were found in the main outcomes evaluated by sex, it must be taken recognized that the target population of the study was not gender-balanced. Patients with chronic disease, caregivers and professionals from health and social sectors are mostly women [38]. That there are women in each of these situations implies a series of particular additional conditions, such as being simultaneously a patient and a caregiver, or being a professional and a caregiver at the same time. In addition, women are at higher risk of poverty during retirement and endure chronic conditions and poorer quality of life for longer than men [39,40]. The results obtained in this study reflect how women do indeed have a worse quality of life, more dependency and receive fewer hours of care from relatives compared with men. Finally, this study has revealed the great need for integrated care in chronic patients for the identification and management of cases able to benefit from the proposed model and receive the most effective social and community services, as shown by the large number of patients assigned with dependency and allocated in daycare centers.

Finally, this study has revealed the urgent need for integrated care for chronic patients in order to identify and manage cases that could be incorporated into this model and receive the necessary social and community benefits. According to the latest report about the evaluation of integrated care in Catalonia, the average aggregate score of the measured indicators was 1.4 on a scale of 0 to 5, and the global score for the quality of social and health care at home obtained a score of 2.7 out of 10. The report highlights that the main barriers to integration are the lack of a culture of, and leadership in, integration at the macro level, the lack of protocols and the lack of shared knowledge in the health and social sectors, as well the lack of shared information systems [41]. In this sense, the results obtained from the *Salut+Social* intervention have proved their value, as evidenced by the substantial number of chronic patients who have been recognized with different degrees of dependency and allocated in daycare centers during the intervention period compared with during previous periods [42].

### Limitations and future research

This study provides compelling information about the effectiveness of integrated care in the improvement of the quality of life and management of chronic patients. However, the study does have some limitations. First, very few PC centers were included. Additionally, recruiting the sample at convenience is almost certain to have introduced a selection bias into the study. Notably, an aged and chronically ill sample shows the expected high rate of attrition due to *exitus* and the impaired capability to be able to participate. Also, the adoption of a new model of a coordinated workforce and the use of new technology requires an initial training and learning effort that is time-consuming and resource-intensive. Thus, the full operation and capacity of the new model may not be realized until a period of adaption has taken place. However, the new technology developed here outweighs the initial effort, as indicated by the positive health and social outcomes achieved.

Further research should be taken in consideration in terms of implementation expansibility, cost-effectiveness and outcomes evaluation. Firstly, the outcomes obtained and the app suitability should be corroborated in a larger sample and region. A cost-effectiveness study should be performed since there is no a stablished or fixed model, thus a further evaluation of the different models implemented is of major relevance for a major efficient use of resources. As well, additional variables and outcomes should be incorporated in future studies in order to provide a more exhaustive, standardized and comparable evaluation.

## Conclusion

Innovation in evidence-based approaches is essential to improve population-based health strategies for chronic disease management. The local/regional setting shown here adds evidence to global health data relevance, hence this approach may be applied in community- based, multidisciplinary and multisectoral healthcare systems to prevent and manage chronic disease. Our results reinforce the claim that models of integrated care may facilitate access to care services, increase perceived patient quality of life and treatment adherence, even though no improved quality of care is recognized. Enhanced access to medical and social services from chronic and socially dependent patients may have important implications for caregivers and care service workers who are struggling to cope with expanding demand.

## Abbreviations

ADL: Activities of daily living; App: Application; DSM-V: Diagnostic and Statistical Manual of Mental Disorders, Fifth Edition; EuroQoL-5D: European quality of life-5 dimensions; GP: General Practitioners; ICT: Information and communication technology; MMAS: Morisky-Green medication adherence scale; PC: Primary Care; PCC: Primary care center.
